# Deceiving and Being Deceived

**Published:** 1858-03

**Authors:** 


### HALL'S JOURNAL OF HEALTH.

OUR LEGITIMATE SCOPE IS ALMOST BOUNDLESS: FOR WHATEVER BEGETS PLEASURABLE AND HARMLESS FEELINGS, PROMOTES HEALTH; AND WHATEVER INDUCES DISAGREEABLE SENSATIONS, ENGENDERS DISEASE.

*We aim to show how Disease may be avoided, and that it is best, when sickness comes, to take no Medicine without consulting an educated Physician.*

VOL. VI.] MARCH, 1858. [No. 3.

### "DECEIVING AND BEING DECEIVED."

Among the very few exchanges which we are neither willing to give away nor cut up, are: *The Phrenological Journal*, *The Water-Cure Journal*, and *Life Illustrated*, published in this city, by Fowler & Wells, 308 Broadway. We look for them, because we always find in them valuable knowledge, new and practical, and well worthy of preservation. The index to one of these volumes, would inspire almost any man of intelligence with a desire to possess them. Every now and then a new view of things is projected, an advance-step made towards what is right and true, and in advance, too, of some who consider themselves far wiser and better than their neighbors. One of these steps was taken some months ago, partially—and we presume is nearing its completion—requiring a considerable degree of moral courage, because it touched the pocket, *the*, THE, THE touchstone of a man's principles; to wit: *Life Illustrated* determined to exclude advertisements from its columns; the reason being, that there was so much deception, downright falsehood, and immorality in them, that it was not safe reading for a family. Can the *Religious Press* afford to exclude these same kinds of advertisements—of gift-book lotteries; of information where men can lend money at twenty-five per cent, forgetting to tell the sequel, that money thus loaned stays loaned; of all sorts of medicines promising all sorts of cures, which promises none know so well are fallacious, as the editors and publishers of these papers? *Life Illustrated* prospers without the aid of these unblushing falsehoods; and so does that very excellent paper, *The American Presbyterian*, of Philadelphia, scarce two years old, and the *Southern Presbyterian*.

We do not wish to exclude *The Religious Press* from the profits of advertising; for the advertising of what is useful, provided it is truthful, is an advantage to any family, and in many instances will cause a saving of time and money, which will pay the subscription-price a dozen times over. When a man wants an article, it is of great advantage to him to know: first, where he can get it; second, that it is a good article; third, that it is at a moderate price. The rule we would lay down is this: Advertise only what is really useful; state where it may be had, and at what price; and throw all adjectives beyond the positive degree into the fire—for instance:

"JAMES THOMAS sells good hats at No. 13 Main street, for three dollars."

"*Hall's Journal of Health*, in good binding, is sold at No. 42 Irving Place, New York, for one dollar and a quarter.

"*The American Agriculturist*, a monthly periodical, is furnished, at one dollar a year, by O. JUDD, 189 Water street, New York."

"*The Young Men's Magazine* is published every month, by R. C. MCCORMICK, 346 Broadway, at a dollar and a half a year —designed to improve the young."

"GOOD ready-made Clothing is sold wholesale, for the Southern market, by DWIGHT & TROWBRIDGE, Chamber-st., New York."

Let us all come down to this Quaker-like simplicity and matter-of-fact truthfulness, and within a single year, a more substantial prosperity will pervade this whole land, in every department of business.

Short weight, short measure, short change, meet us on every side. What doctor, in purchasing a drachm of morphine, ever gets sixty grains? Where is the wholesale house, in New York, that will guarantee that each bolt of cloth will have the full number of yards? Where does the flour-dealer live who sells two hundred pounds of flour for a barrel? Who ever heard of a grocer who sold a pound of candles, single or by the box, for a pound? Is there a shop or store of any description, in all New York, where the odd half or quarter cent has not been unjustly kept by the seller? or at how many places will a child or woman receive in change gold for gold, or city bills for city bills? Are there three importing houses in New York who have always given the Custom House its full legal dues? How many books are printed in this city, the title-pages of which do not convey a falsehood? And it may well be questioned if any law, State or Federal, is ever passed without a bribe. This country is rapidly catching up with Spain and Russia, where no public business is ever thought of being attended to without extra official compensations, and despotic rule cannot put it down; the Czar himself has not the moral courage or confidence to make even a trial! There was significance in the question which Pilate put to the Man Immortal: *What is Truth?* He might have meant, Where is it to be found? Whatever may be its definition, its application, or its place, one thing is certain, its results are, *safety* and HAPPINESS: the happiness of a conscience at rest, exemption from innumerable apprehensions and alarms, all comprehended in the suggestive phrase: "*The Truth shall make you free.*" No wonder is it that a day does not pass in which some attempted or completed suicide is not chronicled—Suicide! the great panacea—how mistaken the thought!—for remorse! the remorse for wrongs done to ourselves or others—wrongs which are always in some way or other connected with untruth, with deception, with "DECEIVING or BEING DECEIVED."

But when a physician has discovered a wrong in the system, he eagerly looks around him for a remedy, and then how to apply it. So we in this case do but practice our profession, in proposing a remedy, and the more radical it is the better. The remedy is not in the "State," nor in the "Church"—both united are utterly powerless to make a truthful people; it is to be accomplished by the mothers of our children: the work is to be begun within a month of birth, and resolutely prosecuted until the years of majority. This sentiment is so self-evident to every well-ordered mind, that we will not enlarge upon it. We do not mean to say, that the father has nothing to do in the moral training of his children; he should take his proper share; but he has to provide for the physical wants of his family, this keeps him from home for the much larger part of his waking existence, and the cares, and annoyances, and fatigues of business, make it impracticable for the majority of men to take a large part in the intellectual and moral education of their children.

It requires no great amount of perspicacity to see the connection between falsehood and disease, between morals and health, between the condition of the mind and the condition of the body, how one involves the other. Remorse makes most of our suicides—remorse drives multitudes every year to a drunkard's premature grave; and how many it sends to that place more dreary than the grave itself, the insane asylum !

But look at the brighter side of the picture. A man of truth is not sad: he may be serious, but it is the seriousness of a pure joy. To be truthful in all we do, or think, or say, in all our actions, and in all intents and purposes, that is none other than "*a well of water springing up into everlasting life*"—a life lasting here, and *ever*lasting hereafter, because it gives serenity of mind, it brings exemption from shocking surprises, from wasting apprehensions, from withering fears, from bootless sorrow; it is the realization, in this life, of the lines familiar to many of us from infancy, written by glorious ISAAC WATTS :

"THE day glides sweetly o'er their heads,
  Made up of innocence and love,
And soft and silent as the shades,
  Their nightly moments gently move.
Swift as their thoughts their joys come on,
  But fly not half so swift away ;
And soft and silent as the shades,
  And calm as summer evenings be."

Another stand which the publishers of *Life Illustrated* have taken, and which has our hearty approval, is that of decided and open condemnation of fictitious reading. This is a matter-of-fact world, and there is more truth in possible fact than in impossible fiction—more poetry and more life. And not more certainly does the drinking of wines, and beers, and brandies destroy the taste for pure cold water, than does fictitious reading take away all relish for realities, for useful and truthful books. A habitual novel-reader, is no more of the world about him than the habitual dram-drinker; neither of them can see things around them as they are; both are living an artificial life, a life unreal, unsatisfactory, and profitless to themselves and to others. And to whatever extent the Religious Press and Religious Publication Societies countenance and foster a taste for fictitious reading, to the same extent do they do a wrong to the great world of Truth, in that they draw away attention from that great Book, whose pages are truth itself.

"Perhaps there is no other country in the world in which light literature is so much encouraged as it is in this. In New York and Boston alone, there are more weekly publications, devoted entirely to productions of this class, than in the whole of the British Empire. Yet all of these publications not only live, but enjoy a wider patronage and a more extensive circulation than the best news-journals of either city can boast of, and should be realizing 'rapid fortunes' for their proprietors, especially as all the half-fledged litterateurs of the day—love-sick young ladies and misanthropical young gentlemen—supply them gratuitously with their lucubrations. What woman or child, capable of reading, is there in this city, who is not accustomed to purchase or borrow some of these papers, and devour their contents with eager interest? Yet what are all these publications made up of, or what is there in them, either in the way of matter or of style, that can entitle them to perusal? Little or nothing; love-stories—love-stories as like each other in incident, style, and denouement as if they were all cut out of the same cloth, as well as made after the same fashion; tales of beautiful blondes, with meek blue eyes, and haughty brunettes, with flashing black ones; and lovers who are all heroes, and poets, and extraordinary genuises.

"Away with all such trash as this, we say! Publications of this kind never did, and never will, answer any useful purpose whatever. On the other hand, they are absolutely injurious in their tendencies; they serve to give those who are addicted to their perusal false views of life—to unsettle the balance of their whole minds, and make them visionary dreamers, instead of earnest doers, in the world.

"The same remarks will also apply to most of the cheap novels of the day, and to the whole of that 'yellow-covered literature' which was of late the rage, though it was the joint product of bile and delirium; and we would scarcely regret it, if all the prose fictions that ever were written were condemned in a lump to the same fate to which the favorite authors of Don Quixote were consigned, for very few of them teach any useful lessons, or contain anything which might not as well go unlearned altogether, or be forgotten as speedily as possible.

"At the best, all fiction, whether prose or verse, is but a luxury of the mind, is unfit for its daily food, and cannot support it in health and vigor. We cannot live on sweatmeats, and would soon die, were we confined to ice-cream and pastries. No father or guardian should ever allow those under his charge to indulge in the habitual reading of 'tales,' if he has any regard for their intellectual or moral health; for they will corrupt the mind and heart as effectually as improper food corrupt the health of the body. But if he see fit to allow the reading of fiction at all, he should assure himself that it does not contain anything objectionable in it, before it goes into their hands, and that it is of the highest stamp of works of this kind. Even in this case, he should remember that the mind which gets to be wedded to novel-reading or tale-reading, soon becomes unfitted for all serious application to necessary and useful studies, and requires the continual stimulus of such exciting matter to fix attention; just as the depraved palate always needs highly seasoned food to give it an appetite."

These sentiments, so well expressed in the January number of the *Phrenological Journal*, will meet the cordial assent of the great mass of reflecting men and women; and we are glad to give them permanency of form in the pages of this Journal. A very near neighbor lady says: that *novel-reading robbed her of all the common sense she ever had.* And we believe it! The result being, she entered real life, with its duties and vast responsibilities, with none at all of that very useful commodity. But she blames her mother for it; and says, she ought to have been switched out of it, and the novels and tales should have been thrown into the fire. Such will be the experience and such the reflection of tens of thousands of daughters, who are now between thirteen and marriage. How many mother-readers of these lines will have the decision, and the energy, and the perseverance, to resolve, that as to their daughters it shall not be so; and not only to resolve it, but conduct it to a successful execution?

The Editor of this Journal allows liberty of sentiment to others, as he hopes to have it allowed to him. He has mentioned three publications in this article with commendation, as being useful to him, as they would be to men and women of matured minds and sentiments; but we do not consider them useful or safe for young people. We would not allow them to be read in our own family, because we consider them, on the whole, as at war against the three great worlds of Medicine, Mind, and Morals. We are an unwavering believer in the truth and virtue of Allopathy; in the power, and necessity, and indispensability of drugs and mineral medicines in the removal of disease and the re-establishment of health. As to morals, we have a conviction as firm as earth's deepest foundations, that the Bible, as interpreted by Evangelical Christianity, is our only *safe* rule of faith and practice. As to the nature and doctrine of the mind, we are satisfied, from sources of information possessed by few, and near opportunities of observation granted not to many, in respect to some of the greatest, the ablest, and the most renowned phrenologists of modern times, that this so-called science, as far as yet expounded, has the effect, either to lodge in, or to produce characters, as utterly destitute of high moral sentiments, as the mind of an untutored savage is destitute of true Christian principles. Mind you, we do not say these things of any persons living in New York, but of persons who live out of our city and State, or are dead. We are not personally acquainted with any phrenologist in this city or State, nor would we know one by sight. We wish to be most distinctly understood, as speaking of the relation between high moral rectitude and phrenology in the character of some eminent phrenologists, whom we personally knew, *and judging from that*, we infer this, and this only, that the GENERAL TENDENCY of phrenology, as it seems to us, is to deteriorate the moral and religious sentiments of those who believe in it. We do not say that there is no truth in phrenology: we know there is truth in it, but it has not yet been so systematized, so reduced, that it can be made a safe chart to sail by; and whether it ever will be so done, we cannot say—the answer is in the future.

Free inquiry is well enough for matured intellects; but to allow a growing family of children free access to newspapers, and periodicals, and books, which are adverse to our own opinions, is a danger which we feel assured, it is not politic to expose our children to; for any man of reflection, of very moderate pretensions to a religious character, must know, that there is no more certain way of making a youth an infidel, than to place in his hands "The Age of Reason."

